# Methodological impact of starch determination on starch content and ileal digestibility of starch in grain legumes for growing pigs

**DOI:** 10.1186/s40104-016-0131-7

**Published:** 2017-01-13

**Authors:** Dagmar Jezierny, Rainer Mosenthin, Nadja Sauer, Klaus Schwadorf, Pia Rosenfelder-Kuon

**Affiliations:** 1 University of Hohenheim, Institute of Animal Science, Emil-Wolff-Strasse 10, 70599 Stuttgart, Germany; 2 Present Address: Present address: Agricultural Analytic and Research Institute Speyer, Obere Langgasse 40, 67346 Speyer, Germany; 3 University of Hohenheim, Core Facility Hohenheim, Emil-Wolff-Strasse 12, 70599 Stuttgart, Germany

**Keywords:** Grain legumes, Growing pigs, Ileal starch digestibility, Starch determination method

## Abstract

**Background:**

Grain legumes represent a valuable energy source in pig diets due to their high starch content. The present study was conducted to determine the content and apparent ileal digestibility (AID) of starch in different grain legume cultivars for pigs by means of both a polarimetric and enzymatic method for starch determination.

**Methods:**

Three experiments were conducted with six barrows each which were fitted with ileal T-cannulas. In total, 18 diets including six different cultivars of faba beans (*Vicia faba L*.) and peas (*Pisum sativum L.*), five different cultivars of lupins (*Lupinus luteus L.*, *Lupinus angustifolius L.*), and one diet with a soybean meal (SBM) were fed.

**Results:**

The starch content of faba beans and peas was greater (*P* < 0.05) when determined polarimetrically than enzymatically (438 vs. 345 g/kg dry matter (DM) in faba beans and 509 vs. 390 g/kg DM in peas, respectively). Considerable lower starch contents were obtained in lupins and SBM, with 82 and 48 g/kg DM (analyzed polarimetrically) and <1.1 and 3 g/kg DM (analyzed enzymatically), respectively. Mean values for contents of neutral detergent fiber (NDF) and acid detergent fiber (ADF) in grain legumes ranged from 111 and 79 g/kg DM in peas to 248 and 207 g/kg DM in lupins, respectively. Contents of condensed tannins in the colored flowered faba bean cultivars ranged from 2.1 to 7.4 g/kg DM. The AID of starch was greater (*P* < 0.05) in pea than in faba bean cultivars, and using the polarimetric starch determination method resulted in greater (*P* < 0.05) digestibility values than using enzymatic starch analysis (84 vs. 80% in faba beans and 86 vs. 83% in peas). Moreover, AID of starch differed (*P* < 0.05) within pea cultivars and starch digestibility in faba beans decreased linearly (*P* < 0.05) as the content of condensed tannins increased. However, there was no relationship between contents of NDF and ADF and AID of starch in pea and faba bean cultivars.

**Conclusion:**

Both contents and AID of starch in grain legumes can vary as influenced by the analytical method used for starch determination. Generally, starch digestibility is greater when measured by polarimetric rather than enzymatic methods.

## Background

The search for alternative protein sources in livestock nutrition has resulted in growing interest in the use of grain legumes as alternatives for animal by-products or oilseed products such as soybean meal (SBM). In addition, grain legumes have proven to be a valuable energy source in animal nutrition due to their high content of starch in faba beans and peas and lipids in lupins [1]. According to NRC [2], average starch contents in faba beans and peas amount to 445 and 493 g/kg dry matter (DM), respectively, which are lower when compared to cereal grains (671, 559, and 708 g/kg DM for wheat, barley, and corn, respectively), but considerably higher than in lupins, SBM, and rapeseed meal (7, 21, and 66 g/kg DM, respectively). The use of grain legumes in livestock nutrition, however, is often limited due to the presence of secondary plant metabolites (tannins, protease inhibitors, alkaloids, lectins, pyrimidine glycosides, saponins), also referred to as anti-nutritional factors (ANF). These metabolites can induce feed refusals (tannins, alkaloids), reduced nutrient digestibility (tannins, protease inhibitors, lectins), or even toxic effects (alkaloids) [3].

In general, legume starch contains 30 to 40% amylose and 60 to 70% amylopectin, whereas cereal starch consists of 20 to 25% amylose and 75 to 80% amylopectin [4]. Hydrolysis of starch by pancreatic *α*-amylase has been reported to be inversely related to the amylose content, with high amylose starches being particularly resistant [5].

As starches in grain legumes are closely associated with proteins, the determination of starch in grain legumes may be confounded by the analytical method used [6], mainly due to the hydrophobic properties of these proteins and the enclosure of the protein-starch network by cell wall compounds [5]. Commonly used procedures for starch determination in food and feed ingredients are based on gravimetric, iodometric, and polarimetric methods [7, 8]. In the European Union, the polarimetric method is the official method used for starch analysis in feeds [9, 10]. According to Champ et al. [11], however, this method lacks precision due to numerous artifacts (e.g. amino acids, mono- and oligosaccharides). Thus, Mitchell [12] suggests the enzymatic method to be advantageous over the polarimetric method for analyzing starch in complex plant matrices such as mixed diets due to enzyme specificity. Until now, no comparative experiments have been conducted to compare values from enzymatic and polarimetric methods for starch determination in different grain legumes and ileal digesta samples collected from pigs fed these grain legumes. Therefore, the objective of the present study was to determine the starch content in different cultivars of faba beans, peas, and lupins and ileal digesta samples by means of a polarimetric and enzymatic procedure. Furthermore, the effect of these different methods as influenced by the presence of ANF in the feed ingredients on apparent ileal digestibility (AID) of starch in growing pigs was assessed.

## Methods

### Experimental procedure

A total of 18 feed ingredients including six seed-grade cultivars of faba beans (*Vicia faba L*.) and peas (*Pisum sativum L*.), five seed-grade cultivars of lupins (*Lupinus L*. spp.), and one commercially available SBM were used in three consecutive experiments with six growing barrows (German Landrace × Piétrain) each. The experiment was arranged as a row-column design. Each of the three experiments included six pigs (rows) and six periods (columns). Within each of the three experiments, the animals were randomly allocated to the 18 assay feed ingredients in periods one to three and four to six, respectively, resulting in two replications per experiment and a total of six observations per assay feed ingredient throughout all three experiments. The average initial and final body weight (BW) of the pigs at the beginning and end of the experiment was 23 ± 2 kg and 45 ± 4 kg, respectively. The pigs were surgically fitted with T-cannulas at the distal ileum according to the procedures described by Li et al. [13]. After a 7-day recuperation period from surgery, the pigs were fed twice daily their semi-synthetic diets in two equal meals (0700 and 1900 h) at a daily level of 30 g/kg (as-fed) of their individual BW, determined on d 1 of every experimental period. Ileal digesta samples were collected for a total of 24 h from 1900 to 0700 h on d 5 and from 0700 to 1900 h on d 6. The individual samples of digesta of each pig were pooled separately after every sampling period, freeze-dried, and ground to 0.5 mm prior to analysis.

The research protocol was approved by the German Ethical Commission for Animal Welfare. Care of the animals used in this experiment was in accordance with the EEC directive 86/609 [14]. The diets based on casein and corn starch contained one of the feed ingredients each (grain legumes or SBM), and were supplemented with (on as-fed basis) 100 g/kg dextrose, 50 g/kg cellulose, 20 g/kg plant oil, and L-cystine, L-threonine, L-lysine-HCl, vitamins, and minerals to fulfil NRC [15] nutrient requirements for pigs from 20 to 50 kg BW. Titanium dioxide was used as a digestibility marker. Further details on diet composition and nutrient contents have been reported by Jezierny et al. [16].

### Analytical procedure

Contents of DM, neutral detergent fiber (NDF), acid detergent fiber (ADF), condensed tannins, and titanium dioxide were determined as described by Jezierny et al. [16].

The starch contents in the feed ingredients, the diets, and in ileal digesta samples were analyzed both polarimetrically and enzymatically. The polarimetric method represents the official starch determination method in the European Union for feed according to the Commission Directive 1999/79/EC [10], and comprises two steps. First, the sample was treated with diluted hydrochloric acid (1.128%). After clarification and filtration, the optical rotation of the solution was measured polarimetrically. In the second step, the sample was extracted with 40% ethanol. After acidifying the filtrate with hydrochloric acid, clarifying, and filtering, the optical rotation was measured according to the same procedure as used in the first step. The difference between these two measurements, multiplied by a factor is equivalent to the starch content of the sample. This factor, however, is not constant as it may vary among various types of starch and feedstuffs. For the quantitative determination of starch by enzymatic analysis, samples had to be pretreated to convert starch into a soluble form. Therefore, homogenized samples were mixed with hydrochloric acid (8 mol/L ) and dimethylsulfoxide and incubated at 60 °C for 30 min. Thereafter, water was added and the solution was adjusted to pH 4–5. After filtration, soluble starch was quantified by enzymatic analysis. For this purpose, starch first was hydrolyzed to D-glucose in the presence of the enzyme amyloglucosidase, followed by phosphorylation of D-glucose to D-glucose-6-phosphate by means of ATP (adenosine-5’-triphosphate) in the presence of hexokinase with simultaneous formation of ADP (adenosine-5’-diphosphate). Then, in the presence of the enzyme glucose-6-phosphate dehydrogenase, the D-glucose-6-phosphate was oxidized by NADP (nicotinamide-adenine dinucleotide phosphate) to D-gluconate-6-phosphate with the formation of reduced NADPH. The amount of NADPH formed is stoichiometric to the amount of D-glucose formed by hydrolysis of starch and was quantified spectrophotometrically at 340 nm (type Lambda 25, PerkinElmer Inc., Waltham, MA) [17].

### Calculations

The AID of starch in the diets was calculated using the following equation:

$$ \mathrm{I}{\mathrm{D}}_{\mathrm{Ai}} = 100\%\ \hbox{--}\ \left[\left({\mathrm{I}}_{\mathrm{Ai}} \times {\mathrm{S}}_{\mathrm{D}\mathrm{i}}\right)\ /\ \left({\mathrm{S}}_{\mathrm{Ai}} \times {\mathrm{I}}_{\mathrm{D}\mathrm{i}}\right)\right] \times 100\% $$

where ID_Ai_ = apparent ileal starch digestibility in the i^th^ diet (%), I_Ai_ = marker concentration in the i^th^ diet (g/kg DM), S_Di_ = starch content in digesta of the i^th^ diet (g/kg DM); S_Ai_ = starch content in the i^th^ diet (g/kg DM), and I_Di_ = marker concentration in digesta of the i^th^ diet (g/kg DM).

The AID of starch in the feed ingredients was calculated according to the following equation:

$$ \mathrm{I}{\mathrm{D}}_{\mathrm{I}} = \left(\mathrm{I}{\mathrm{D}}_{\mathrm{Ai}}\hbox{--}\ \mathrm{I}{\mathrm{D}}_{\mathrm{C}} \times {\mathrm{C}}_{\mathrm{C}}\right)\ /\ {\mathrm{C}}_{\mathrm{I}} $$

where ID_I_ = apparent ileal starch digestibility in the feed ingredient (%), ID_Ai_ = apparent ileal starch digestibility in the diet (%), ID_C_ = apparent ileal digestibility of corn starch (%), C_C_ = contribution level of starch from corn starch to the diet (%) and C_I_ = contribution level of starch from the feed ingredient to the diet (%).

### Statistical analysis

Homogeneity of variances and normal distribution of the data were confirmed and experimental data were analyzed using the MIXED procedure of SAS [18]. The linear model included the fixed effects of grain legume species, experiment, grain legume cultivar, analysis, species × cultivar (cultivar is nested within species), experiment × animal, experiment × replication, species × analysis and the random effects experiment × replicate × period and experiment × replicate × animal. Multiple comparisons among cultivars within species were performed using a *t*-test with degrees of freedom determined by the Kenward-Roger method [19]. This test was performed only when a preliminary *F*-test [20] revealed significant differences within a species (SLICE = species option in MIXED). Significant differences between treatments were indicated by different superscript letters using the algorithm for letter-based representation of all pair-wise comparisons according to Piepho [21]. The significance level was set at α = 0.05. Furthermore, the effects of NDF and ADF contents in all grain legumes and of tannin contents in faba beans on AID of starch were modelled by a linear regression.

## Results

### Chemical composition of grain legumes

Analyzed contents of starch, both determined polarimetrically and enzymatically, and contents of NDF and ADF in grain legume cultivars as well as condensed tannins in faba bean cultivars are presented in Table 1.

**Table 1:** Analyzed contents of carbohydrates and condensed tannins of feed ingredients (g/kg DM)

Species	Cultivar	DM	Starch		NDF	ADF	Condensed tannins
			*polarimetric*	*enzymatic*			
*Pisum sativum*	Santana^1^	881	503	372	105	81	ND
	Jutta^1^	869	505	389	104	79	ND
	Phönix^1^	877	518	392	106	68	ND
	Harnas^1^	871	496	385	109	86	ND
	Rocket^1^	874	495	378	126	83	ND
	Hardy^1^	872	535	423	115	75	ND
*Vicia faba*	Aurelia^1^	878	456	358	126	101	ND
	Divine^2^	883	452	371	128	112	2.1
	Gloria^1^	886	425	314	127	111	ND
	Limbo^2^	890	442	393	138	116	7.0
	Fuego^2^	876	421	321	165	137	7.4
	Espresso^2^	872	432	311	156	134	4.2
*Lupinus* spp.	Probor^3^	902	95	<1.1^5^	224	185	ND
	Bornal^4^	892	56	<1.1^5^	252	208	ND
	Boregine^3^	909	96	<1.1^5^	247	195	ND
	Boruta^3^	901	90	<1.1^5^	261	230	ND
	Idefix^3^	905	74	<1.1^5^	258	219	ND
SBM		905	48	3	114	74	ND

*Abbreviations*: *ADF* acid detergent fiber, *DM* dry matter, *ND* not determined, *NDF* neutral detergent fiber, *SBM* soybean meal

^1^White flowered cultivar

^2^Colored flowered cultivar

^3^ *L. angustifolius*

^4^ *L. luteus*

^5^values were below the detection limit of 1.1 g/kg DM

Within pea and faba bean cultivars, starch contents ranged between 495 and 535 g/kg DM for cultivars Rocket and Hardy, respectively, and between 421 and 456 g/kg DM for cultivars Fuego and Aurelia, respectively, when determined polarimetrically. Using the enzymatic approach, starch contents within pea cultivars ranged from 372 to 423 g/kg DM for cultivars Santana and Hardy, and within faba bean cultivars from 311 to 393 g/kg DM for cultivars Espresso and Limbo. Within lupin cultivars, starch content ranged from 56 in Bornal to 96 g/kg DM in Boregine when determined polarimetrically. However, starch content for all lupin cultivars was below the detection limit of 1.1 g/kg DM when analyzed enzymatically. The starch content in SBM amounted to 48.0 and 3.0 g/kg DM, based on polarimetric and enzymatic measurements, respectively. Contents of NDF and ADF in faba bean cultivars ranged from 126 to 165 and 101 to 137 g/kg DM, respectively, and in the pea cultivars from 104 to 126 and 68 to 86 g/kg DM, respectively. Condensed tannins contents in faba beans amounted to 2.1, 4.2, 7.0, and 7.4 g/kg DM in Divine, Espresso, Limbo, and Fuego, respectively. In peas and lupins, condensed tannins were not detected.

Average starch contents in pea, faba bean, and lupin cultivars are presented in Table 2. Among grain legume species, pea cultivars had greatest average starch contents, followed by faba beans, and lowest contents in lupins, irrespective of the method used for determination (*P* < 0.05). Average starch contents in pea, faba bean, and lupin cultivars amounted to 509, 438, and 82 g/kg DM when analyzed polarimetrically and 390, 345, and <1.1 g/kg DM, respectively, when analyzed enzymatically. The mean polarimetric analyzed starch values in grain legumes were greater when compared to the mean starch contents that were analyzed enzymatically (*P* < 0.001) (Table 2).

**Table 2:** Effect of starch determination method on starch content in grain legumes^#^

	Starch content, g/kg DM		*P*-value
Species	*polarimetric*	*enzymatic*	
*Pisum sativum*	509 ± 8.0^aC^	390 ± 8.0^bC^	<0.001
*Vicia faba*	438 ± 8.0^aB^	345 ± 8.0^bB^	<0.001
*Lupinus* spp.	82 ± 8.8^A^	<1.1^*A^	<0.001

^#^LSmeans ± standard error of the means

*Abbreviations*: *DM* dry matter

^a,b^Within a row, means without a common superscript differ (*P* < 0.05)

^A,B,C^Within a column, means without a common superscript differ (*P* < 0.05)

^*^values were below the detection limit of 1.1 g/kg DM

### Ileal starch digestibility in grain legumes

The AID of starch of faba beans and peas is shown in Table 3 using either polarimetric or enzymatic analysis for starch determination. The AID of starch for lupins and SBM could not be determined by means of the difference method due to their low starch contents, resulting in a low contribution level to the total starch contents in the diet.

**Table 3:** Apparent ileal starch digestibility in faba beans and peas

Species	Cultivar	Ileal starch digestibility (in %)		SEM	*P*-value
		*polarimetric*	*enzymatic*		
*Pisum sativum*	Santana^1^	86^abc^	85^ab^		
	Jutta^1^	89^ab^	88^a^		
	Phönix^1^	83^c^	80^bc^		
	Harnas^1^	89^a^	84^ab^		
	Rocket^1^	83^c^	77^c^		
	Hardy^1^	85^bc^	82^bc^		
	LSmeans	86^d^	83^eB^	0.8	<0.001
*Vicia faba*	Aurelia^1^	86	81		
	Divine^2^	86^*^	82		
	Gloria^1^	85	77		
	Limbo^2^	81^*^	75		
	Fuego^2^	84^*^	80		
	Espresso^2^	85^*^	82		
	LSmeans	84^d^	80^eA^	0.8	<0.001

*Abbreviation*s: *SEM* standard error of the means

^1^White flowered cultivar

^2^Colored flowered cultivar

^a,b,c^Within a column, means without a common superscript differ within species (*P* < 0.05)

^d,e^Within a row, means without a common superscript differ between starch determination methods (*P* < 0.05)

^A,B^Within a column, means without a common superscript differ between species (*P* < 0.05)

^*^linear effect of dietary content of condensed tannins (*P* < 0.05)

Within pea cultivars, AID of starch was greater (*P* < 0.05) for cultivar Harnas (89%) in comparison to cultivars Phönix (83%), Rocket (83%), and Hardy (85%), when the polarimetric method was used for starch analysis. Based on the enzymatic procedure, AID of starch within peas was greater (*P* < 0.05) for cultivar Jutta (88%) when compared to cultivars Hardy (82%), Phönix (80%), and Rocket (77%). Apparent ileal starch digestibility in faba beans did not differ (*P* > 0.05) between cultivars, and ranged from 81 (Limbo) to 86% (Aurelia, Divine) using the polarimetric starch determination method and from 75 (Limbo) to 82% (Divine, Espresso) with the enzymatic starch determination procedure.

Mean values for AID of starch did not differ between faba bean and pea cultivars (84 vs. 86%) when the polarimetric method was used (Table 3). However, average AID of starch was lower (*P* < 0.05) in faba beans when compared to pea cultivars (80 vs. 83%), if starch contents were analyzed enzymatically. Similarly, mean values for AID of starch of faba beans and peas were lower (*P* < 0.05) when starch content was determined enzymatically rather than polarimetrically.

## Discussion

Mean values for polarimetrically analyzed starch contents of peas were within the range of previously reported data [22, 23]. Based on this approach, starch contents in faba beans were greater and starch contents in blue lupins lower when compared to values reported by Berk et al. [23] and Moschini et al. [22]. However, the polarimetric method resulted in a starch content of 56 g/kg DM in the yellow lupin cultivar Bornal which was greater than average values published by Berk et al. [23] for yellow lupins (35 g/kg DM). Starch content of SBM was in good agreement with tabulated values published by DLG [24], which have been determined polarimetrically as this procedure is used as official method for feed analysis in Germany [7]. Using the enzymatic starch determination procedure, average starch contents in the grain legumes were consistently lower when compared to literature data, with up to 111, 160, and 12 g/kg DM lower contents for peas [25–27], faba beans [25, 28], and lupins [29], respectively. Comparative studies on measurements of starch contents in grain legumes by using polarimetric and enzymatic procedures have not been published, but the results reported herein were confirmed by Obuchowski et al. [30] in different triticale grain samples. The authors determined up to 109 g/kg DM lower starch contents when using the enzymatic method rather than the polarimetric procedure. According to Beutler [8], results obtained by the polarimetric method may be biased by the presence of substances, such as mono-, oligo-, and polysaccharides or fiber in feed and food ingredients. Thus, greater starch values obtained by the use of the polarimetric method can be attributed to the fact that both starch and other carbohydrates such as pentosans and *ß*-glucans are hydrolyzed [30]. Similarly, Priepke et al. [29] reported that measurement of starch contents in lupins according to the polarimetric approach were confounded by the presence of mono-, oligo-, and polysaccharides. The enzymatic starch analysis method, however, is based on the principle that starch is hydrolyzed to glucose. Complete hydrolysis of starch to glucose and specific measurement of the released glucose are required to obtain accurate starch values. However, incomplete hydrolysis of starch to glucose or incomplete assay of released glucose due to inadequate gelatinization or conditions that reduce enzymatic activity, will result in lower starch values when the enzymatic method is used [6].

Differences in starch contents within grain legume cultivars may be due to differences in genetics, but also to varying growing and harvesting conditions [31, 32]. Jørgensen et al. [33] and Prolla et al. [34], however, observed no differences in starch content of peas and common beans, respectively, between different years of harvest, Therefore, it cannot be ruled out that in the present study both variations in harvest and growing conditions and genetics may be responsible for the differences in starch content within grain legume cultivars.

In the present study, AID of starch of faba beans was up to 13 percentage units lower compared to AID values obtained by Jansman et al. [35] in piglets. Results may be biased by the use of different methods for starch analysis as these authors used the polarimetric and enzymatic starch determination method for feed and digesta samples, respectively. For raw peas, AID of starch determined with the enzymatic method was up to 13 percentage units lower in comparison with results by Stein and Bohlke [36] who reported AID of starch of 90% in raw peas using the same analytical procedure. However, in a study with growing pigs using an enzymatic procedure for starch determination, Sun et al. [37] obtained AID of starch coefficients between 78 and 80% in raw peas, measured in different experimental periods. These values were lower than those for most pea cultivars in the present study.

Hydrolysis of starch in the gastrointestinal tract is influenced by a number of host factors, in addition to differences in physical characteristics of the ingested food and starch. Host factors, for example, include intensity of chewing the feed, the availability of pancreatic α-amylases, and digesta retention time in the small intestine [38]. Starch depolymerization is affected by several digestive enzymes that cleave the α-(1–4-) and α-(1–6-) glycosidic bonds. In monogastric species, the main starch hydrolyzing enzymes are α-amylases, amyloglucosidases, and isoamylases [39, 40]. There are differences in starch digestibility between different categories of feed ingredients such as digestibility of starch in peas in comparison to e.g. cereal starch is lower at the ileal level (*P* < 0.05), but there are no differences over the entire digestive tract [41]. Consequently, more starch from peas than from cereals will enter the large intestine. According to Wiseman [41], up to 20% of starch digestion from peas will take place in the large intestine compared with only 4 to 7% for cereal starch.

Differences in AID of starch have been attributed to the existence of an enzyme-resistant starch fraction, differences in starch digestion rate, or both [42]. It is generally acknowledged that starch digestibility is affected by the crystal structure of amylopectin present in starch granules [42]. Accordingly, A-type pattern found in cereal starches have proven to be highly digestible, whereas B-type pattern exhibited by starches from tubers, such as potatoes, renders them more resistant to digestion by pancreatic amylase; and C-type pattern found in grain legume starches being intermediate between the A- and B-type patterns revealing some resistance to hydrolysis by α-amylases [37, 43, 44]. Gernat et al. [45] hypothesized that the C-type pattern is a mixture of the A- and B-type patterns with varying ratios. According to these authors, pea starch is composed of 61.4% type A and 38.6% type B, in comparison to starch of beans with 83.0% type A and 17.0% type B. However, these structural differences in starch granules among grain legumes do not explain the greater AID of starch in peas when compared to faba beans in the present study. Besides variations in crystallinity of amylopectin, the amylose content is another important factor affecting starch digestibility [46, 47]. Unlike amylopectin that is highly branched, amylose polymers have less surface area and more intramolecular hydrogen bonds [42]. Therefore, amylose is depolymerized at a slower rate and extent than amylopectin due to decreased accessibility for α-amylase [48]. According to Sun et al. [37], digestibility of starch is generally inversely proportional to its amylose content, because the amylase starts hydrolyzing in the amorphous regions of the amylopectin [49]. However, these findings could not be confirmed by the results of the present study, where faba beans exhibited lower digestibility values than peas despite greater amylose contents in peas (44.4%) than in faba beans (40.0%) [50]. According to the results of a recently conducted literature review [44], there is little information on the structure of grain legume starches at different levels of structural organization (granular, supramolecular, and molecular). Consequently, it is difficult in many instances to explain the variation in characteristics among grain legume starches reported in the literature. Moreover, there is limited information on grain legume starches with respect to susceptibility towards acid and enzyme hydrolysis, polymorphic composition, rate and extent of retrogradation, and on the levels of rapidly digestible starch, slowly digestible starch, and resistant starch. As many of the studies on grain legume starches have been conducted on individual cultivars, it is difficult to conclude, whether characteristics of structure and their relationships reported for an individual cultivar are truly representative for the whole species [44]. Finally, it has to be acknowledged that particle size may also contribute to differences in starch digestibility among feed ingredients of the same origin. For example, according to Owsley et al. [51], reduction in particle size of sorghum resulted in improved apparent ileal and total digestibility of DM, starch, protein, and GE of growing pigs.

Grain legume starches are generally part of the protein matrix [5]. Most of these proteins are quite hydrophobic, and the protein-starch network is surrounded by cell walls. The starch tends therefore to be kept in the interior of the particles protected from water. Starches from tubers and legumes are particularly well protected from the polar environment of luminal fluids [5] resulting in limited access of host enzymes including α-amylase.

Differences in AID of starch between pea cultivars in the present study may also be attributed to the presence of cell wall components surrounding the starch granules, thereby inhibiting access of amylases to the granule. According to results of in vitro digestion of starch in red kidney beans (*Phaseolus vulgaris*), amylolysis was enhanced by wet homogenization and pepsin pretreatment [52]. These findings suggest that disruption of cell walls is important for efficient starch digestion, however, no relationship between AID of starch and NDF or ADF contents in the diets could be established (data not shown), likely due to low variation among cultivars.

In faba beans, condensed tannins reduce AID of starch in pigs (e.g. [53]). Condensed tannins strongly inhibit the activity of α-amylase, maltase, sucrase, and lactase [54]. As these enzymes are the most important carbohydrases, condensed tannins may interfere with in vivo carbohydrate digestion and absorption, thus increasing the proportion of starch reaching the large intestine. This is in accordance with the results of the present study, where the AID of starch (determined polarimetrically) of the tannin containing faba bean cultivars Divine, Espresso, Limbo, and Fuego decreased (*P* < 0.05) with increasing tannin content of the diets (amounting to 0.9, 1.8, 2.7, and 3.0 g/kg DM for diets containing the cultivars Divine, Limbo, Fuego, and Espresso, respectively). Differences between AID of starch in faba beans and peas or within pea cultivars may be attributed to variations in contents of resistant starch. Moreover, variations in amylose to amylopectin ratio of grain legumes may, at least in part, explain the observed differences in AID of starch.

## Conclusions

Starch contents and AID of starch in grain legumes obtained by means of the polarimetric method are greater than starch values determined enzymatically. This methodical difference is apparently due to the presence of other feed components such as carbohydrates that interfere with the polarimetric starch analysis and (or) has to be attributed to incomplete starch hydrolysis when the enzymatic procedure is applied. Differences in AID of starch between faba beans and peas are confined to the use of the enzymatic method for starch determination. Within pea cultivars, differences in AID of starch were observed for both starch analysis methods. There was a negative linear effect of condensed tannins in the diet on AID of starch in faba beans when the polarimetric method was used for starch determination. The polarimetric approach is the official method used in the European Union for starch determination in feed. However, there is increasing evidence that the enzymatic method is superior over the polarimetric method due to its enzyme specificity for starch.

## Abbreviations

ADF: Acid detergent fiber
AID: Apparent ileal digestibility
ANF: Anti-nutritional factors
BW: Body weight
DM: Dry matter
NDF: Neutral detergent fiber
SBM: Soybean meal
